# Glyphosate Induces Liver Macrophage Pyroptosis via Mitochondrial Damage-Mediated cGAS-STING Activation

**DOI:** 10.3390/toxics14060461

**Published:** 2026-05-25

**Authors:** Xiangyu Yu, Jiawen Ren, Ying Kang, Shizhi Wang, Jianrui Dou, Yongquan Yu

**Affiliations:** 1 Key Laboratory of Environmental Medicine Engineering, Ministry of Education, School of Public Health, Southeast University, Nanjing 210009, China; yxy000923@163.com (X.Y.); rjw67516@163.com (J.R.); 220254161@seu.edu.cn (Y.K.); shizhiwang2009@seu.edu.cn (S.W.); 2 Yangzhou Center for Disease Control and Prevention, Yangzhou 225007, China

**Keywords:** glyphosate, NHANES, liver injury, macrophage, pyroptosis, cGAS-STING

## Abstract

Glyphosate, the most widely used herbicide worldwide, is now ubiquitous in the environment, posing a growing threat to human health. While accumulating evidence has linked glyphosate exposure to liver injury, the underlying mechanisms remain unclear. In this study, based on data from NHANES 2013–2018, we identified significant associations between glyphosate exposure and abnormal liver function parameters in the general US population. A glyphosate-exposed mouse model was further established, and the results showed that hepatic accumulation of glyphosate induced direct histopathological damage and increased serum AST, ALT, and ALP levels in mice. Combined network toxicology and gene set analyses revealed that glyphosate activated liver macrophages, upregulating genes related to lipid metabolism, inflammation, and pyroptosis. The activation of the pyroptosis pathway was further confirmed by Western blot analysis of NLRP3 inflammasome-associated proteins. Mechanistically, glyphosate disrupted mitochondrial membranes and compromised mitochondrial function, leading to the release of mtDNA, which subsequently activated the cGAS-STING pathway in mouse livers and RAW264.7 macrophages. Moreover, glyphosate-induced NLRP3 activation in RAW264.7 cells was attenuated by the cGAS inhibitor. These findings provide a novel mechanistic insight into glyphosate-induced hepatotoxicity and reinforce the growing concern over its association with liver injury in humans.

## 1. Introduction

Glyphosate is a broad-spectrum pesticide that exerts its herbicidal effect by inhibiting the shikimate pathway, a plant-specific metabolic route essential for aromatic amino acid biosynthesis [1,2]. Since its introduction in the 1970s, and particularly following the widespread adoption of genetically modified glyphosate-resistant crops in the mid-1990s, its global usage has increased dramatically, with global glyphosate usage projected to reach approximately 920 thousand tonnes by 2025 [3]. This massive and sustained use has led to pervasive environmental contamination [4,5,6], making glyphosate and its primary metabolite aminomethylphosphonic acid (AMPA) detectable in soil [7], water [8], and food systems [9]. Consequently, human exposure is widespread. Studies have consistently detected glyphosate in urine samples across diverse populations. In the United States, a cohort of 71 pregnant women in Indiana (2015–2016) showed a detection rate of 93%, with mean urinary concentrations of 3.4 μg/L, and notably higher levels in rural residents (4.19 μg/L) compared to suburban/urban subgroups (3.17–3.47 μg/L) [10]. Similarly, in non-farming households in Iowa, glyphosate was detectable in 88% of children (mean 2.5 μg/L) and 65% of mothers (mean 1.2 μg/L) [11]. Widespread glyphosate exposure has also been reported across Europe. Notably, biomonitoring in France detected the herbicide in over 99% of urine samples from the general population, while substantial exposure levels have also been observed in Germany, Switzerland, and Iceland [12,13]. Despite its plant-specific mechanism of action, which has historically classified glyphosate as low-risk for mammals [14], accumulating evidence has raised concerns over its potential genotoxicity [15,16], endocrine disruption [17], and hepatotoxicity [18,19], sparking scientific debate and underscoring the need to clarify the molecular mechanisms underlying glyphosate-induced health effects.

The liver serves as a central metabolic hub and one of the first tissues to encounter environmental toxicants absorbed through the intestine, making it uniquely susceptible to xenobiotics such as glyphosate [20]. This vulnerability is underscored by recent experimental evidence across diverse model systems, which demonstrates that glyphosate induces hepatotoxicity, leading to histopathological lesions, oxidative stress, and inflammatory responses in the liver [21,22,23]. Yet, the mechanisms underlying glyphosate-induced liver injury, particularly how it triggers hepatic inflammation, remain to be elucidated. There is growing evidence that glyphosate can induce inflammatory responses in the liver [24,25], with elevated pro-inflammatory cytokines and disrupted immune homeostasis reported in animal models. At the cellular level, this inflammatory response is closely related to the liver’s resident macrophages, which account for roughly 90% of the body’s total macrophage population and act as key sentinels in sensing xenobiotic challenge [26]. Liver capsular macrophages (LCMs) were activated after GLY exposure as reflected by the significantly increased cell proportion and upregulation of phagocytosis, endocytosis, and inflammatory response. Given that the liver is the primary site for the accumulation and metabolism of environmental chemicals, persistent glyphosate exposure may exploit macrophage-mediated inflammatory pathways, thereby contributing to the onset or progression of hepatic dysfunction [27]. Elucidating how glyphosate engages liver macrophages to orchestrate inflammatory responses thus represents a crucial step toward understanding its hepatotoxic potential.

Pyroptosis is a form of inflammatory cell death driven by gasdermin-mediated membrane pore formation, which facilitates the release of pro-inflammatory cytokines and intracellular contents [28]. This process is orchestrated by the NLRP3 inflammasome, which, once activated, recruits ASC and pro-caspase-1 to drive caspase-1 autoactivation. The active caspase-1 then cleaves gasdermin D (GSDMD) to generate its pore-forming fragment while also processing IL-1β and IL-18 into their mature forms [29]. A key upstream trigger for NLRP3 inflammasome activation is mitochondrial damage, which leads to mtDNA release into the cytosol and subsequent activation of the cGAS-STING pathway, a key innate immune sensor. cGAS-STING signaling can, in turn, promote NLRP3 inflammasome assembly and pyroptosis [30]. Notably, glyphosate exposure has been shown to disrupt mitochondrial function and activate NLRP3/caspase-1 signaling in renal and glioblastoma cells, suggesting a conserved mechanism that may extend to the liver [31,32]. However, whether glyphosate induces pyroptosis in liver macrophages via mtDNA-mediated cGAS-STING activation remains unclear. Elucidating this pathway is therefore essential for understanding the hepatotoxic potential of glyphosate.

In this study, we first investigated the association between glyphosate exposure and liver function in humans using data from the National Health and Nutrition Examination Survey (NHANES). A chronic exposure model was then established in mice to assess glyphosate distribution in the liver, along with its effects on liver enzyme functions and histopathological changes. Integrated network toxicology and gene set analyses were performed to explore the underlying mechanisms, with a focus on liver macrophage pyroptosis. In vitro experiments using RAW264.7 cells further examined the involvement of mitochondrial damage-mediated cGAS-STING activation in glyphosate-induced pyroptosis. Collectively, this work aims at elucidating the molecular mechanisms by which glyphosate contributes to liver injury in mice and providing evidence-based insights into its hepatotoxic effects on liver health.

## 2. Materials and Methods

### 2.1. Study Design and Population

**Figure 1:**
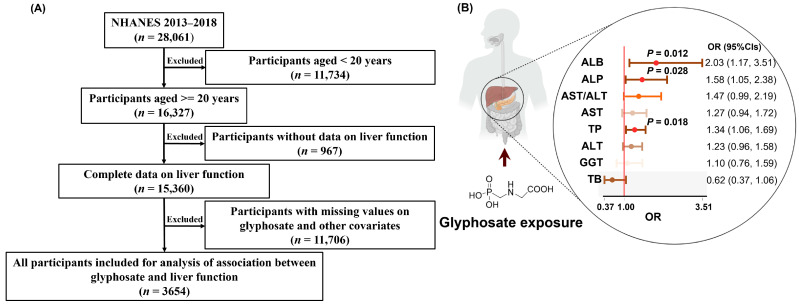
Association between urinary glyphosate and liver function outcomes. (**A**) Schematic diagram of participant enrollment in the study (NHANES 2013–2018). (**B**) Survey-weighted logistic regression for the association between urinary glyphosate and liver function outcomes. Line segments marked with red dots indicate statistical significance.

NHANES is a nationally representative, cross-sectional survey conducted by the National Center for Health Statistics (NCHS) to assess the health and nutritional status of the non-institutionalized US population. It integrates questionnaire data, physical examinations, and laboratory measurements, providing a comprehensive resource for studying environmental exposures and health outcomes [33]. In this study, a total of 16,327 participants aged ≥ 20 years were initially identified from the NHANES 2013–2018 cycles. After excluding individuals with missing data on glyphosate exposure, liver function outcomes, and relevant covariates (*n* = 12,673), the final study comprised 3654 participants for analysis. All protocols were approved by the NCHS Research Ethics Review Board, and participants provided written informed consent. Details regarding participant selection are depicted in Figure 1A.

Specifically, glyphosate exposure for each individual was estimated based on urinary concentrations, as measured by 2D online ion chromatography–tandem mass spectrometry coupled with isotope dilution quantification (lower limit of detection [LLOD] = 0.200 ng/mL). Eight indicators of liver function, namely, alanine aminotransferase (ALT), aspartate aminotransferase (AST), alkaline phosphatase (ALP), gamma-glutamyl transferase (GGT), albumin (ALB), total protein (TP), total bilirubin (TB), and the AST/ALT ratio, were selected to evaluate liver health. Abnormal liver function was defined based on previously recommended cutoff values: ALT > 40 U/L [34], AST > 40 U/L [34], ALP > 128 U/L [35], GGT > 61 U/L [36], ALB < 3.4 g/dL [36], TP < 6.5 g/dL [37], TB > 1.2 mg/dL [38], or AST/ALT ratio ≥ 2 [39].

### 2.2. Animal Models

The animal experiment protocol was approved by the Ethics Committee of Southeast University (20231128020), and detailed procedures were described in our previous publication [40]. A total of 40 male C57BL/6 mice (8 weeks old, 20–25 g) were obtained from GemPharmatech (Nanjing, China) and housed at the Experimental Animal Center of Southeast University. All mice were bred in ventilated cages (5 mice per cage) under stable environmental conditions with a 12 h day/night rhythm at ambient temperature (25 °C  ±  1 °C) and humidity range (50–60%). Normal diet and clean water were available ad libitum. After one week of acclimatization, mice were randomly assigned to four groups (n = 10 per group), namely, control (saline, C), low-dose (glyphosate, 2 mg/kg/day, L), medium-dose (20 mg/kg/day, M), and high-dose (200 mg/kg/day, H). Glyphosate was administered by gavage for 12 consecutive weeks (6 days/week). Glyphosate (purity ≥ 99.5%) was obtained from Aladdin (Shanghai, China). Doses were selected to cover a range from environmentally relevant to toxicologic levels: the low dose was based on the EPA reference dose, while the medium and high doses were set at 10- and 100-fold multiples, respectively, with all doses remaining below the no observable adverse effect limit (500 mg/kg/day) for systemic toxicity. At the study endpoint, mice were sacrificed by cervical dislocation, and liver tissues, serum (separated by centrifugation at 3000 rpm for 10 min), and urine were harvested and stored at −80 °C until analysis.

### 2.3. Glyphosate and AMPA Levels in Mouse Liver and Urine

Glyphosate and AMPA in liver and urine were quantified using LC–MS/MS analysis based on a previous study [41]. Briefly, for liver samples, 50 mg of tissue was homogenized in 0.5 mL of Milli-Q water and centrifuged at 4 °C, 12,000× *g* for 10 min, and the supernatant was collected; for urine samples, 10 μL of urine was diluted with 90 μL of Milli-Q water. Aliquots (100 μL) of liver supernatant or diluted urine were mixed with isotopically labeled internal standards (^13^C_2_, ^15^N-glyphosate and ^13^C_2_, ^15^N-AMPA), followed by the addition of EDTA solution and acetonitrile; vortexed; centrifuged; and subjected to phase separation by freezing at −20 °C, with the lower aqueous phase collected for instrumental analysis. Chromatographic separation was performed on an ACQUITY Premier UPLC system using a gradient elution program with mobile phases consisting of 2.5% formic acid in water containing 10 mmol/L of a passivating agent and acetonitrile. Mass spectrometric analysis was conducted using a triple quadrupole mass spectrometer (Agilent 1290LC-6460, Agilent Technologies, Santa Clara, CA, USA). The LLOD was 4 ng/g for glyphosate in liver and 0.4 ng/mL in urine and 3 ng/g for AMPA in liver and 0.3 ng/mL in urine.

### 2.4. Biochemical Analysis

Serum biochemical parameters related to liver function and lipid metabolism were measured on an automatic biochemical analyzer (Hitachi, Osaka, Japan) following the manufacturer’s protocols. Briefly, the levels of ALT and AST were determined using kinetic methods, while ALP was measured via the NPP substrate–AIP buffer method. Total cholesterol (CHO) and triglycerides (TG) were quantified using the PAP method, high-density lipoprotein cholesterol (HDL-C) and low-density lipoprotein cholesterol (LDL-C) were assessed by a direct homogeneous method, and non-esterified fatty acids (NEFAs) were measured using the ACS–ACOD method.

### 2.5. Histological Analysis

Liver tissues were collected and processed for histological evaluation with paraffin-embedded and frozen sections. For hematoxylin and eosin (H&E) staining, liver specimens were fixed in 4% paraformaldehyde for 24 h, dehydrated through an alcohol gradient, cleared in xylene, infiltrated with paraffin, and embedded. Sections of 5 μm thickness were then stained with H&E solutions following standard protocols and imaged using a digital scanning system (3DHISTECH, Budapest, Hungary). For Oil Red O staining, fresh liver tissues were embedded in OCT tissue freezing medium (SAKURA, Tokyo, Japan), snap-frozen at −80 °C, and sectioned using a cryostat. Frozen sections were stained with Oil Red O solution in the dark for 10 min, differentiated in 60% isopropanol, rinsed with water, counterstained with hematoxylin, and then subjected to bluing and final washing steps before imaging using the digital scanning system (3DHISTECH, Budapest, Hungary).

### 2.6. Network Toxicology Analysis

To explore the molecular basis of glyphosate-induced hepatotoxicity, a network toxicology approach was conducted. The structure of glyphosate was obtained from the PubChem database, and potential protein targets were predicted using three complementary platforms, namely, ChEMBL 36, Swiss Target Prediction 2019, and TargetNet v1.0.0, with a probability score threshold ≥ 0.5. The species were restricted to “Mus musculus” and “Rattus norvegicus”. The filtering parameters were set as |logFC| ≥ 1, *p* < 0.05, and FDR < 0.05. Targets associated with liver injury were collected from the Comparative Toxicogenomics Database (CTD, 2024 release; https://ctdbase.org/; accessed 17 March 2026), and a filtering threshold of Inference Score > 50 was applied to focus on high-confidence targets. Intersection analysis between glyphosate-associated and liver injury-related datasets was performed using R 4.5.2 with the ggvenn and viridis packages, and the overlapping targets were visualized using a Venn diagram. Functional enrichment analysis was conducted using the clusterProfiler package in R 4.5.2, based on the Gene Ontology (GO) and Kyoto Encyclopedia of Genes and Genomes (KEGG) databases. The statistical thresholds were set as *p* < 0.05 and, after FDR/Bonferroni correction, *p* < 0.01.

### 2.7. Quantitative PCR (qPCR) Analysis

Total RNA was isolated from mouse liver samples using TRIzol reagent (Vazyme, Nanjing, China). RNA concentration and purity were assessed by measuring the absorbance at 260/280 nm using a NanoDrop spectrophotometer. Reverse transcription was carried out with a commercial kit (Vazyme, Nanjing, China) to synthesize complementary DNA (cDNA) following the manufacturer’s instructions. qPCR analysis was performed using SYBR Green Master Mix (Vazyme, Nanjing, China) on a QuantStudio 7 Flex Real-Time PCR system (Thermo Fisher, Waltham, MA, USA). The cycling conditions were set according to the standard protocol provided with the master mix. Relative gene expression levels were calculated using the 2^−ΔΔCt^ method, with β-actin serving as the endogenous control. Primer sequences used for amplification are listed in Table S1.

### 2.8. Cell Culture and Treatment

The mouse mononuclear macrophage cell line RAW264.7 was used as a model for investigating macrophage-mediated responses. Cells were cultured in high-glucose Dulbecco’s modified Eagle’s medium (DMEM) supplemented with 10% fetal bovine serum (FBS) and 1% penicillin–streptomycin and maintained at 37 °C in a humidified atmosphere containing 5% CO_2_. For cytotoxicity assessment, cells were seeded in 96-well plates and exposed to a range of glyphosate concentrations (0.1–10 mM) for 24 h, after which cell viability was evaluated using the CCK-8 kit (Beyotime, Shanghai, China). Based on the cell viability assay, glyphosate exposure for 24 h induced a significant decrease in cell viability, beginning at 2 mM. Thus, 2 mM was designated as the low dose, while 5 mM (the next concentration showing incremental effects) was used as the high dose in follow-up studies (Figure S2). For mechanistic analysis, cells were seeded in 6-well plates and cultured overnight prior to treatment. Cells were then exposed to glyphosate at 2 (Concentration Low, CL) and 5 mM (Concentration High, CH) for 24 h, either alone or in the presence of the cGAS inhibitor C176 (1 µM; MedChem Express, Monmouth Junction, NJ, USA).

### 2.9. Transmission Electron Microscopy (TEM) Analysis

The organelle ultrastructure of RAW264.7 cells was investigated using TEM. Following the designated treatment, cells were gently detached by trypsinization and immediately fixed in 2.5% glutaraldehyde. After rinsing with PBS, samples were post-fixed in 1% osmium tetroxide, dehydrated through a graded ethanol series, and infiltrated with epoxy resin prior to embedding and polymerization. Ultrathin sections were then prepared, double-stained with uranyl acetate and lead citrate, and examined under a TEM (JEOL, Tokyo, Japan), with representative images captured for analysis.

### 2.10. DCFH-DA and JC-1 Staining

Intracellular reactive oxygen species (ROS) production in RAW264.7 cells was evaluated using the DCFH-DA probe (Beyotime, Shanghai, China). Following experimental treatments, cells were harvested, washed twice with PBS, and incubated with DCFH-DA in serum-free medium for 20 min at 37 °C in the dark. After rinsing twice with PBS to remove excess probe, ROS fluorescence intensity was measured by flow cytometry (Cytek Biosciences, Fremont, CA, USA), with at least 10,000 events acquired per sample.

Mitochondrial membrane potential (MMP) was assessed using the JC-1 fluorescent dye (Beyotime, Shanghai, China). After the designated treatments, RAW264.7 cells were incubated with JC-1 working solution for 30 min at 37 °C in the dark, followed by two washes with PBS to remove unbound dye. Fluorescence signals, representing JC-1 aggregates (red) and monomers (green), were visualized under a fluorescence microscope (ZEISS, Oberkochen, Germany), with representative images captured for analysis.

### 2.11. Measurement of Intracellular ATP Content

Intracellular ATP content was determined using a commercial luciferin–luciferase assay kit (Beyotime, Shanghai, China) in accordance with the provided protocol. Briefly, following the designated treatment, RAW264.7 cells were lysed with RIPA lysis buffer, centrifuged at 12,000× *g* for 5 min at 4 °C, and the supernatants were collected. For detection, 100 μL of ATP assay working solution was pre-dispensed into each well and incubated at room temperature for 3–5 min to deplete background ATP. Subsequently, 20 μL of sample or standard was added and mixed rapidly, and the relative luminescence units (RLU) were recorded using a luminometer (BERTHOLD, Bad Wildbad, Germany).

### 2.12. Measurement of Cytoplasmic mtDNA

Cytosolic mtDNA levels in RAW264.7 cells were assessed after subcellular fractionation. Briefly, cells were trypsinized, washed with PBS, and collected by centrifugation (100–200× *g*, 5–10 min, room temperature). After resuspension in ice-cold PBS, the cell suspension was centrifuged again (200× *g*, 5 min, 4 °C). The pellet was resuspended in 1 mL of mitochondrial isolation buffer A (Beyotime, Shanghai, China) containing PMSF, vortexed gently, and incubated on ice for 10–15 min. Cells were homogenized in a glass homogenizer, and the homogenate was centrifuged at 1200× *g* for 10 min at 4 °C. The supernatant was transferred to a fresh tube and centrifuged again at 12,000× *g* for 10 min at 4 °C; the final supernatant was collected as the cytosolic fraction. DNA was extracted using a TIANamp Genomic DNA Kit (TIANGEN, Beijing, China), and mtDNA abundance was quantified by qPCR analysis.

### 2.13. Western Blot Analysis

Total protein was extracted from mouse liver tissues and RAW264.7 cells using RIPA lysis buffer supplemented with protease and phosphatase inhibitors. After lysis on ice for 30 min with vortexing every 10 min, lysates were centrifuged at 12,000× *g* for 15 min at 4 °C, and supernatants were collected. Proteins were then separated by SDS-PAGE and transferred onto PVDF membranes. After blocking with 5% non-fat dry milk, membranes were incubated with primary antibodies overnight at 4 °C, followed by incubation with HRP-conjugated secondary antibodies for 1–2 h at room temperature. GAPDH was used as an internal control. Protein bands were imaged with a chemiluminescence detection system. Band intensities were quantified using ImageJ (Version 1.54g) software. Primary antibodies used are listed in Table S2.

### 2.14. Statistics Analysis

For the analysis of epidemiological data, continuous variables were summarized as mean ± standard deviation (SD), while categorical variables were reported as frequencies and percentages. Urinary glyphosate concentrations were log-transformed to normalize their distribution prior to analysis. Associations between glyphosate exposure and liver function outcomes (ALT, AST, ALP, GGT, ALB, TP, TBIL, and AST/ALT ratio) were assessed using survey-weighted logistic regression models, accounting for the complex sampling design and subsample weights of the NHANES. Results were presented as odds ratios (ORs) with 95% confidence intervals (CIs). All models were adjusted for urinary creatinine, as well as sociodemographic factors (sex, race/ethnicity, poverty income ratio [PIR], and education level), lifestyle behaviors (body mass index (BMI), alcohol consumption, physical activity, and smoking exposure), and history of cardiovascular disease (CVD).

For the analysis of experimental data, all results were expressed as mean ± SD. Statistical analyses were performed using SPSS software (version 17.0). Differences among multiple groups were assessed using one-way or two-way analysis of variance (ANOVA), followed by post hoc tests: least significant difference (LSD) when variances were homogeneous or Games–Howell when variances were heterogeneous. A *p*-value of less than 0.05 was considered statistically significant.

## 3. Results

### 3.1. Association Between Urinary Glyphosate and Liver Function Outcomes

A total of 3654 participants from the NHANES 2013–2018 cycles were included for final analysis. The mean age of the study population was 50.05 years, with males and females accounting for 49.7% and 50.3%, respectively. Almost half of participants were non-Hispanic White (41.7%), and approximately 79.4% were at or above the poverty level. More than one-third of individuals had received some college or associate degree education. The average urinary glyphosate concentration was 0.49 ng/mL, with a detection rate of 76.1%. Further demographic details are summarized in Table S3. Using survey-weighted logistic regression models adjusted for all covariates, glyphosate exposure was significantly associated with several indicators of liver dysfunction (Figure 1B). Higher urinary glyphosate levels were positively associated with elevated odds of abnormal ALB (OR: 2.03, 95%CI: 1.17 to 3.51), ALP (OR: 1.58, 95%CI: 1.05 to 2.38), and TP (OR: 1.34, 95%CI: 1.06 to 1.69). No significant associations were observed between glyphosate and other liver function parameters. These findings provide evidence linking higher urinary glyphosate levels to compromised liver function among the studied population.

### 3.2. Chronic Glyphosate Exposure Induces Liver Injury in Mice

**Figure 2:**
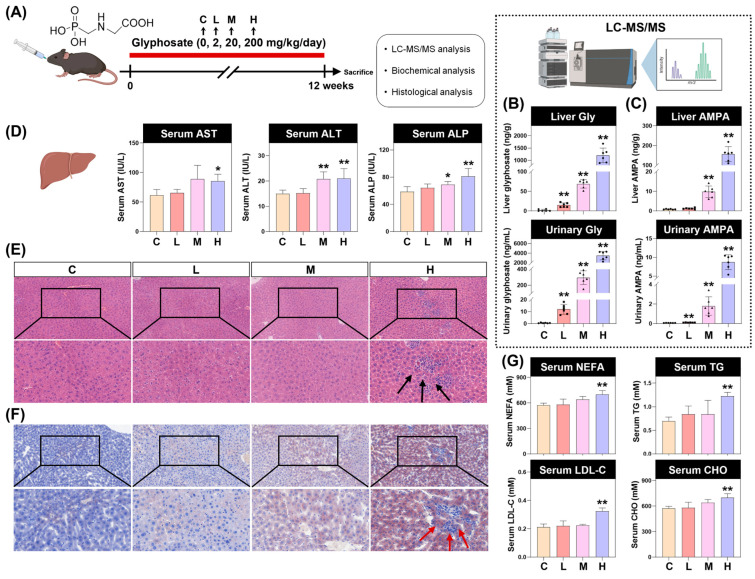
Chronic glyphosate exposure induces liver injury in mice. (**A**) Experimental scheme of glyphosate exposure in vivo. (**B**,**C**) Quantification of glyphosate (**B**) and AMPA (**C**) in mouse liver and urine samples (n = 6). (**D**) Measurement of serum AST, ALT, and ALP levels (n = 6). (**E**,**F**) Representative images of liver sections stained with (**E**) H&E and (**F**) Oil Red O. Scale bars: 50 μm (top panel); 20 μm (bottom panel, magnified view). Black arrows indicate inflammatory infiltration. Red arrows indicate the presence of lipid droplets. More details are provided in Figure S5. (**G**) Measurement of serum NEFA, TG, LDL-C, and CHO (n = 5). Data are expressed as mean ± SD. * *p* < 0.05 and ** *p* < 0.01, compared with the control group.

A mouse model was established in which C57BL/6J mice received glyphosate (2, 20, or 200 mg/kg/day) or vehicle control by gavage for 16 weeks (Figure 2A). Internal exposure was assessed using an optimized LC-MS/MS method for quantifying glyphosate and its metabolite AMPA in urine and liver tissues, with calibration curves showing excellent linearity (R^2^ > 0.99) (Figure S1). A dose-dependent increase in both glyphosate and AMPA concentrations was observed in urine and liver tissues of treated mice (Figure 2B,C), confirming successful systemic exposure.

Liver function indicators and histopathological analysis revealed pronounced hepatic injury following glyphosate exposure. Specifically, serum AST, ALT, and ALP levels were significantly elevated in the medium- and high-dose groups (Figure 2D). H&E staining showed significant inflammatory infiltrate in the high-dose group (Figure 2E), whereas Oil Red O staining revealed marked, dose-dependent hepatic lipid accumulation (Figure 2F). Moreover, markers of lipid metabolism, including NEFA, TG, LDL-C, and TC, were all significantly elevated in the high-dose group (Figure 2G). Collectively, these findings indicate that glyphosate exposure triggers hepatic inflammation, disrupts lipid homeostasis, and leads to significant liver dysfunction in mice.

### 3.3. Glyphosate Activates Pyroptosis-Mediated Inflammatory Response in Mouse Livers and RAW264.7 Macrophages

**Figure 3:**
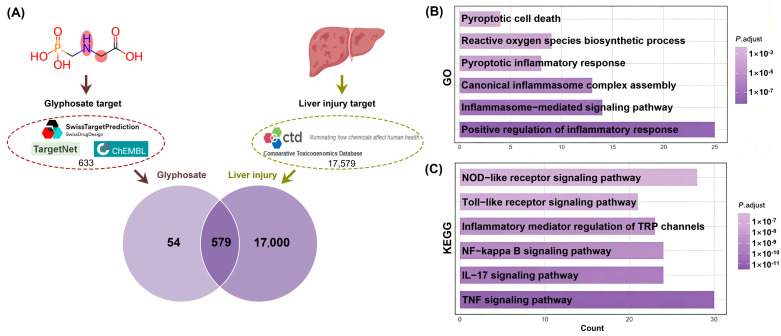
Network toxicology analysis of glyphosate-induced liver injury. (**A**) Venn diagram of glyphosate targets and liver injury targets. Brown arrows indicate the process of identifying potential targets of glyphosate. Green arrows indicate the process of identifying liver injury-related targets. (**B**) GO and (**C**) KEGG enrichment analyses of 579 targets implicated in glyphosate-related liver injury.

**Figure 4:**
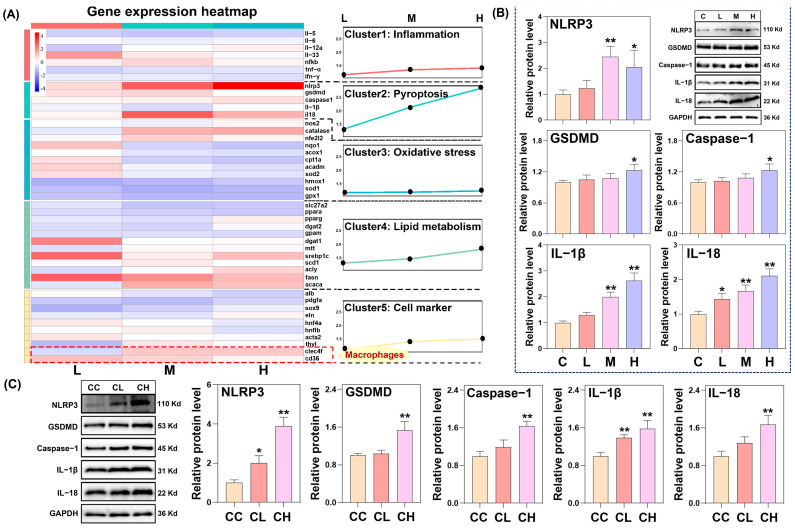
Glyphosate activates macrophages and induces inflammation and NLRP3-mediated pyroptosis in mouse liver and RAW264.7 cells. (**A**) Heatmap analysis of genes involved in inflammation, pyroptosis, oxidative stress, lipid metabolism, and hepatic cell markers; the Y-axis of the cluster plot denotes the normalized expression levels of the indicated gene groups. Black dashed lines separate the genes into five functional clusters, which correspond to the trend plots shown on the right: Cluster 1, Inflammation; Cluster 2, Pyroptosis; Cluster 3, Oxidative stress; Cluster 4, Lipid metabolism; and Cluster 5, Cell markers. The red dashed rectangle highlights the macrophage marker genes Clec4f and Cd36. (n = 3). (**B**,**C**) Expression of NLRP3 inflammasome-associated proteins in (**B**) mouse liver and (**C**) RAW264.7 cells (n = 3). Data are expressed as mean ± SD. * *p* < 0.05, ** *p* < 0.01, compared with the control group.

To explore the molecular basis of glyphosate-induced hepatotoxicity, potential targets associated with glyphosate exposure and liver injury were retrieved from public databases, with 633 and 17,579 candidates identified, respectively (Figure 3A). Overlap analysis between the two datasets was performed using a Venn diagram, resulting in 579 shared targets implicated in glyphosate-related liver damage (Figure 3A). Functional analysis of these common targets through GO enrichment highlighted significant involvement in inflammatory responses, pyroptosis, and oxidative stress (Figure 3B). Consistently, KEGG pathway mapping pointed to the TNF, IL-17, and NF-κB signaling axes as key pathways underlying glyphosate hepatotoxicity (Figure 3C). These predictions were further validated by qPCR analysis in liver tissues from glyphosate-exposed mice. As shown in Figure 4A, glyphosate treatment induced a pro-inflammatory state, characterized by upregulated pro-inflammatory cytokines and oxidative stress, along with enhanced expression of lipid metabolism-related genes. Notably, pyroptosis-associated genes, including *nlrp3*, *gsdmd*, *caspase-1*, *il-1β*, and *il-18*, were consistently elevated. Moreover, significant upregulation of *cd36* and *clec4f* was observed across hepatic cell populations, indicating macrophage activation. The activation of the pyroptosis pathway was also confirmed by Western blot analysis of pyroptosis-associated proteins (NLRP3, GSDMD, Caspase-1, IL-1β, and IL-18) in mouse livers and RAW264.7 macrophages (Figure 4B,C). Taken together, these findings suggest that glyphosate-induced liver injury may stem from macrophage-mediated inflammatory responses and pyroptosis.

### 3.4. Mitochondrial Damage-Mediated cGAS-STING Activation Is Involved in Glyphosate-Induced Pyroptosis in Macrophages

**Figure 5:**
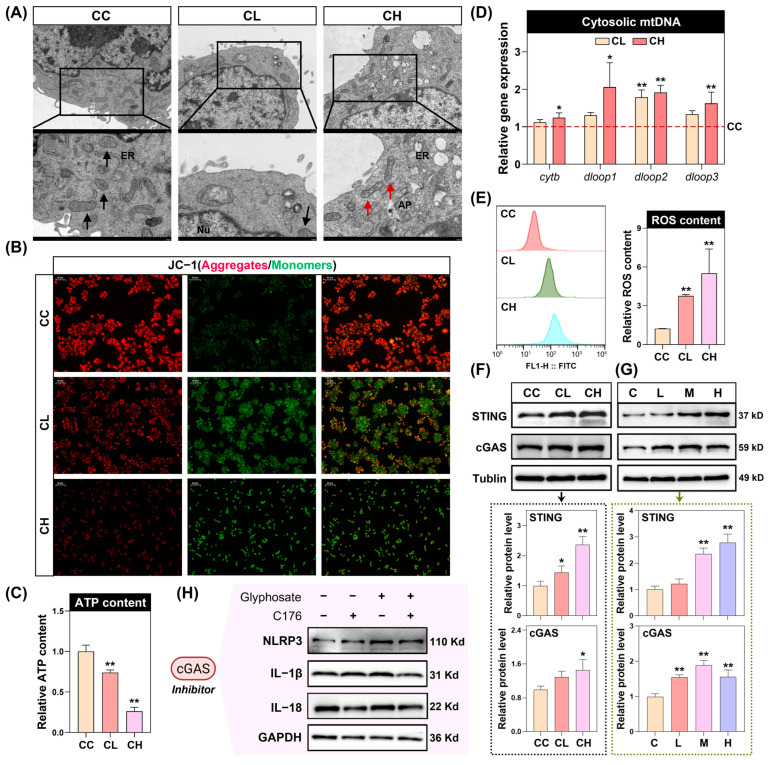
Glyphosate impairs mitochondria and induces the mtDNA-cGAS-STING pathway to activate NLRP3-mediated pyroptosis in macrophages. (**A**) Mitochondrial ultrastructure in RAW264.7 cells was assessed by TEM. Nu, nuclear; ER, endoplasmic reticulum; AP, autophagosome. Scale bars: 1 μm (top panel); 0.5 μm (bottom panel, magnified view). Black arrows indicate normal mitochondria; red arrows indicate impaired mitochondria: membrane lysis, along with disarray and fragmentation of the cristae. More details are provided in Figure S6. (**B**) Representative fluorescence images of MMP in RAW264.7 cells. Red fluorescence indicates polarized (healthy) mitochondria, whereas green fluorescence represents depolarized mitochondria. Scale bars: 40 μm. (**C**) Relative ATP content in RAW264.7 cells (n = 3). (**D**) Quantification of mtDNA abundance in the cytosol of RAW264.7 cells using qPCR (n = 3). (**E**) Quantification of intracellular ROS in RAW264.7 cells via flow cytometry (n = 3). (**F**,**G**) Expression of cGAS-STING in (**F**) mouse liver and (**G**) RAW264.7 cells (n = 3). (**H**) Expression of NLRP3 inflammasome-associated proteins in RAW264.7 cells (n = 3). Data are expressed as mean ± SD. * *p* < 0.05 and ** *p* < 0.01, compared with the control group.

To elucidate the upstream mechanism of pyroptosis activation in macrophages, we next examined whether glyphosate activates the cGAS-STING pathway, a cytosolic DNA sensor that is triggered upon mitochondrial damage and subsequent mtDNA release. As shown in Figure 5A, cells in the control group exhibited intact mitochondria with well-preserved cristae and clearly defined membranes. In contrast, exposure to glyphosate led to blurred membranes, disorganized or fragmented cristae, and vacuolization, indicating marked mitochondrial injury. Moreover, glyphosate exposure induced dose-dependent reductions in ATP production and MMP, along with increased ROS levels, indicating mitochondrial dysfunction in RAW264.7 cells (Figure 5B,C,E). To assess mtDNA release, mitochondrial and cytosolic fractions were isolated. Cytosolic levels of mtDNA components (nd1, nd4, cytb, dloop1, dloop2, and dloop3) were significantly elevated following glyphosate treatment (Figure 5D). Consistent with these findings, protein expression of cGAS and STING was upregulated in both liver tissues and RAW264.7 cells (Figure 5F,G). The functional involvement of the cGAS-STING pathway in glyphosate-induced pyroptosis was further corroborated using the cGAS inhibitor C176, which markedly attenuated the elevation of NLRP3 inflammasome-related proteins (NLRP3, IL-1β, and IL-18) (Figure 5H). Collectively, these data suggest that glyphosate disrupts macrophage mitochondria, thereby driving mtDNA-dependent cGAS-STING pathway activation and subsequent pyroptosis.

## 4. Discussion

In this study, a population-based analysis using nationally representative NHANES data was coupled with in vivo experimentation to establish the association between glyphosate exposure and liver injury and elucidate its underlying mechanistic basis. Epidemiological findings revealed significant positive associations between urinary glyphosate levels and abnormal liver function parameters in general US adults. In the complementary mouse model, chronic glyphosate exposure resulted in direct hepatic histopathological damage, hepatic lipid accumulation, and elevated serum liver enzymes, recapitulating the human findings. Mechanistically, glyphosate disrupted mitochondrial membrane integrity and compromised mitochondrial function, leading to the release of mtDNA, which subsequently activated the cGAS-STING pathway in mouse livers and RAW264.7 macrophages. This cascade further promoted macrophage pyroptosis, ultimately contributing to glyphosate-induced hepatotoxicity. These findings establish a novel mechanistic link between glyphosate exposure and liver injury, highlighting the role of the mtDNA-cGAS-STING–pyroptosis axis in glyphosate-associated hepatotoxicity.

As the most widely applied herbicide worldwide, glyphosate has been extensively documented as inducing toxicity in multiple organs, with the liver being one of the major targets due to its central role in xenobiotic metabolism [42,43]. Despite accumulating concerns regarding its hepatotoxic potential, population-based evidence linking glyphosate exposure to liver injury remains scarce [44]. Recent cross-sectional studies utilizing NHANES data have reported positive associations between urinary glyphosate exposure and compromised liver function, liver fibrosis, and non-alcoholic fatty liver disease [45,46,47]. A prospective cohort study further reported that childhood glyphosate exposure was associated with elevated liver transaminases and metabolic syndrome in young adults, providing longitudinal evidence supporting an early-life window of susceptibility [46]. However, these studies have been largely constrained by reliance on limited survey cycles or restriction to specific subpopulations. In the present study, we extended these findings by leveraging nationally representative data from NHANES 2013–2018, encompassing three survey cycles, and observed significant positive associations between urinary glyphosate concentrations and multiple liver function biomarkers, including ALB, ALP, and TP, after accounting for survey weights and adjusting for potential confounders. By incorporating a larger and more diverse population sample, our findings provide further epidemiological evidence supporting the association between environmental glyphosate exposure and liver injury in the general US adult population.

Glyphosate is pervasive in the environment and is routinely absorbed through inhalation, dermal contact, and digestion, among which ingestion via consumption of contaminated water and food constitutes the primary route for individual exposure [48]. Therefore, to explore the mechanisms underlying the observed positive association between glyphosate exposure and compromised liver function in the general population, an in vivo study was conducted in which glyphosate was administered via gavage at doses based on the EPA reference dose. This exposure regimen allows glyphosate and its metabolite AMPA to enter the liver, suggesting that glyphosate may exert damaging effects on the liver. After carefully reviewing the EPA official guideline, the chronic reference dose (cRfD) for glyphosate is 1.75 mg/kg/day. Based on this, the low dose was set at 2 mg/kg/day to simulate environmental exposure levels. The medium-dose (20 mg/kg/day) and high-dose (200 mg/kg/day) groups were set at 10- and 100-fold multiples of the cRfD, respectively, to evaluate dose-dependent effects and identify potential molecular mechanisms that may not be detectable at lower exposure levels. Importantly, the highest dose (200 mg/kg/day) remains below the no-observed-adverse-effect level (NOAEL) for systemic toxicity in mice (500 mg/kg/day), ensuring that the observed effects are not simply due to overt toxicity. Although the medium and high doses exceed typical human environmental exposure levels, they are necessary to establish causal relationships and mechanistic pathways in animal models, which can then inform risk assessment for environmentally relevant exposures. Moreover, to further contextualize our animal findings within the framework of human exposure, we measured urinary glyphosate concentrations in the mouse model and found that the mean level in the low-dose group (11.96 ng/mL) was comparable to the value observed in the NHANES population (8.21 ng/mL in maximum), highlighting the translational relevance of our model. Consistent evidence from rodent studies has demonstrated histopathological damage and elevated serum liver enzymes following glyphosate exposure [49]. Supporting this, our results revealed that glyphosate exposure elicited a dose-dependent and induced significant inflammatory infiltration in the liver, accompanied by dose-dependent increases in serum AST, ALT, LDL−C and TG levels (Figure S4). Furthermore, increased hepatic inflammation was evidenced by combined network toxicology and gene set analyses, which identified pyroptosis and macrophage activation following glyphosate exposure. Collectively, these findings implicate macrophage-mediated hepatic pyroptosis and inflammation as drivers of glyphosate-induced liver injury.

Hepatic macrophages are central to the initiation and persistence of liver inflammation, functioning as key effectors of the innate immune response [50]. Under pathological conditions, their polarization toward a pro-inflammatory M1 phenotype disrupts the homeostatic balance between the M1 subset and the anti-inflammatory M2 subset, leading to sustained release of inflammatory mediators [51]. This imbalance not only amplifies local inflammation but also creates a permissive microenvironment for progressive liver injury. A critical molecular pathway underlying this process involves the assembly of the NLRP3 inflammasome, which is triggered by damage-associated molecular patterns (DAMPs) released from stressed or injured cells. The assembly of NLRP3 with the adaptor ASC and procaspase-1 generates a multimeric complex that facilitates the maturation of pro-inflammatory cytokines and promotes pyroptosis, a form of pro-inflammatory programmed cell death [52,53]. Pyroptosis further exacerbates inflammation by releasing intracellular contents, including DAMPs, thereby establishing a self-amplifying cycle that sustains macrophage activation and tissue injury. Previous studies have demonstrated that glyphosate can induce NLRP3 inflammasome-mediated pyroptosis in immune cells such as microglia and neutrophils [40]. In line with these studies, our results reveal that glyphosate significantly increased the expression of pyroptosis-associated proteins, including NLRP3, GSDMD, Caspase-1, IL-1β, and IL-18, in both mouse liver tissue and RAW264.7 macrophages, confirming this mechanism in liver macrophages. However, how glyphosate activates NLRP3 inflammasome-mediated pyroptosis in macrophages remains to be elucidated.

The cGAS-STING signaling cascade has emerged as a critical regulator of NLRP3 inflammasome-mediated pyroptosis, exerting control over both its priming and activation phases. Upon engagement, STING coordinates the activation of transcription factors, including NF-κB, which drives the expression of inflammasome components, a process commonly identified as priming [54]. In addition, STING signaling can induce lysosomal membrane permeabilization and potassium ion efflux, providing the requisite secondary signals for inflammasome complex assembly [55]. Through this synergistic mechanism, the cGAS-STING axis serves as an important upstream modulator bridging early priming events with the subsequent activation of NLRP3 [56]. Supporting this, we found that glyphosate treatment elicited a dose-dependent upregulation of cGAS and STING expression, an effect observed in both in vivo and in vitro models. Furthermore, pharmacological inhibition of cGAS with the selective inhibitor C176 markedly suppressed glyphosate-induced NLRP3 inflammasome signaling, corroborating that the cGAS-STING axis mediates the pyroptotic response triggered by glyphosate. It has been reported that mitochondrial dysfunction represents an important route leading to cGAS-STING activation. Disruption of mitochondrial homeostasis can trigger the translocation of mitochondrial DNA to the cytosol, where it serves as a DAMP recognized by cGAS [57]. In line with this mechanism, glyphosate exposure induced mitochondrial ultrastructural damage in macrophages, including cristae fragmentation and membrane disruption, accompanied by functional deficits such as reduced ATP synthesis, elevated ROS, and loss of MMP. This organellar dysfunction led to cytosolic release of mtDNA, which acted as a DAMP to engage the cGAS-STING signaling axis. Furthermore, the cGAS inhibitor C176 was used to validate the above mechanism. Quantitative protein analysis showed that, compared with the control group, C176 treatment alone did not result in statistically significant changes in NLRP3 or IL-1β protein levels. In contrast, glyphosate alone significantly increased the expression of these inflammatory proteins. Moreover, compared with glyphosate alone, combined treatment with C176 and glyphosate significantly attenuated the glyphosate-induced upregulation of inflammatory protein expression (Figure S3). Taken together, these findings indicate that glyphosate triggers mitochondrial dysfunction, leading to cytosolic mtDNA release and subsequent cGAS-STING activation, which in turn orchestrates the activation of NLRP3 inflammasome-mediated pyroptosis in macrophages.

## 5. Conclusions

In summary, this study integrates epidemiological evidence with experimental models to show that glyphosate exposure is associated with liver injury. NHANES data revealed positive associations between urinary glyphosate levels and liver function biomarkers, findings that were recapitulated in mice exhibiting glyphosate-induced hepatic damage, steatosis, and elevated transaminases. Mechanistically, glyphosate triggered mitochondrial dysfunction in macrophages, leading to cytosolic release of mtDNA and subsequent activation of the cGAS-STING pathway, which orchestrated the activation of the NLRP3 inflammasome and ultimately promoted pyroptotic cell death. These findings provide potential public health implications for mitigating glyphosate-related liver injury, particularly given the widespread use of this herbicide worldwide.

## Data Availability

The raw data supporting the conclusions of this article will be made available by the authors upon request.

## Supplementary Materials

The following supporting information can be downloaded at https://www.mdpi.com/article/10.3390/toxics14060461/s1: Figure S1: Quantitative LC-MS/MS analysis of glyphosate and AMPA; Figure S2: Assessment of RAW264.7 cell viability following glyphosate exposure; Figure S3: Quantification of NLRP3-related proteins after glyphosate and C176 treatment; Figure S4: Spearman correlation between urine/serum compound and liver Gly/AMPK content; Figure S5: Representative images of liver sections stained with (A) H&E and (B) Oil Red O; Figure S6: Mitochondrial ultrastructure in RAW264.7 cells was assessed by TEM; Table S1: List of oligonucleotide primer sequences; Table S2: Primary antibodies used in the present work; Table S3: Baseline characteristics of participants in NHANES 2013–2018.

## Abbreviations

The following abbreviations are used in this manuscript:

AMPA	Aminomethylphosphonic acid
GSDMD	Gasdermin D
mtDNA	Mitochondrial deoxyribonucleic acid
NHANES	National Health and Nutrition Examination Survey
NCHS	National Center for Health Statistics
LC–MS/MS	Liquid chromatography–tandem mass spectrometry
LLOD	Lower limit of detection
ALT	Alanine aminotransferase
AST	Aspartate aminotransferase
ALP	Alkaline phosphatase
GGT	Gamma-glutamyl transferase
ALB	Albumin
TP	Total protein
TB	Total bilirubin
CHO	Cholesterol
TG	Triglyceride
HDL-C	High-density lipoprotein cholesterol
LDL-C	Low-density lipoprotein cholesterol
NEFAs	Non-esterified fatty acids
H&E	Hematoxylin and eosin
GO	Gene Ontology
KEGG	Kyoto Encyclopedia of Genes and Genomes
qPCR	Quantitative polymerase chain reaction
TEM	Transmission electron microscopy
ROS	Reactive oxygen species
MMP	Mitochondrial membrane potential
RLU	Relative luminescence units
SD	Standard deviation
OR	Odds ratio
CI	Confidence interval
PIR	Poverty income ratio
BMI	Body mass index
CVD	Cardiovascular disease
ANOVA	Analysis of variance
LSD	Least significant difference
DAMP	Damage-associated molecular pattern
