# Supplementary material for: Glyphosate Induces Liver Macrophage Pyroptosis via Mitochondrial Damage-Mediated cGAS-STING Activation
Source: Toxics. 2026 May 25;14(6):461. doi: 10.3390/toxics14060461 (PMC13306997; doi:10.3390/toxics14060461)
Supplement: Supplementary file 1 [file toxics-14-00461-s001.zip › toxics-4270158-supplementary.pdf]

Glyphosate Induces Liver Macrophage Pyroptosis via Mitochondrial Damage-mediated cGAS-STING Activation

Supplemental data

Table of Contents

Table S1. List of oligonucleotide primer sequences.

Table S2. Primary antibodies used in the present work.

Table S3. Baseline characteristics of participants in NHANES 2013–2018.

Figure S1. Quantitative LC-MS/MS analysis of glyphosate and AMPA.

Figure S2. Assessment of RAW264.7 cell viability following glyphosate exposure.

Figure S3. Quantification of NLRP3-related proteins after glyphosate and C176 treatment.

Figure S4. Spearman correlation between urine/serum compound and liver Gly/AMPK content.

Figure S5. Representative images of liver sections stained with (A) H&E and (B) Oil Red O.

Figure S6. Mitochondrial ultrastructure in RAW264.7 cells was assessed by TEM.

Table S1. List of oligonucleotide primer sequences.

Gene	Forward 5'->3' primera	Reverse 5'->3' primer
β-actin	GGCTGTATTCCCCTCCATCG	CCAGTTGGTAACAATGCCATGT
nlrp3	ATTACCCGCCCGAGAAACTT	TCATGCAGCCCAGATTCTCT
gsdmd	CCTTGGGTGAGGTTCTGAAG	GCATCTCCAGACTGGTTCTG
caspase1	ATGGCATCTTCATGAGCACAG	TCAGCTTCAATGTCCTCATCA
il-1β	GCAACTGTTCCTGAACTCAACT	ATCTTTTGGGGTCCGTCAACT
il-18	ACTGTACGACGCTTCGGTG	TCCTCTTACCTGAAGCCGTT
mt-Cytb	CCATCCAACATCTCAGCATGATGAAA	GCCCATTTCAGGTTCATAGTCC
mt-Dloop 1	CACCATTAGCACCCAAAGCT	TGGTGGAATGGGTTTTATGG
mt-Dloop 2	CTCACGGGAGCTCTCCATGC	CGGCTGTGGCAGGTTT
mt-Dloop 3	GCCCCATGCATTTGGTAACA	GCCCCATGCATTTGGTAACA
il-5	AGGCTTCCTGTCCCTACTCAT	AGTACCCCCACGGACAGTTT
il-6	AGACAAAGCCAGAGTCCTTCAG	TGTGACTCCAGCTTATCTCTTGG
il-12a	CCTGCTGAAGACCACAGATGAC	GCACAGGGTCATCATCAAAGAC
il-33	TCCAACTCCAAGATTTCCCCG	TTATGGTGAGGCCAGAACGG
nfkb	CCCTACGGAACTGGGCAAAT	GCGGAATCGAAATCCCCTCT
tnf-α	CGGGCAGGTCTACTTTGGAG	ACCCTGAGCCATAATCCCCT
ifn-γ	GAGGTCAACAACCCACAGGT	GGGACAATCTCTTCCCCACC
nos2	GCCAACATGCTACTGGAGGT	TGGAGCACAGCCACATTGAT
catalase	GAAGGACCGTGTTTGGTTGC	CCGCTGGCGCTTTTATTGTT
nfe212	AGAGTGATGGTTGCCCACTT	GCGTGCTCAGAAACCTCCTT
nqo1	TAGCCTGTAGCCAGCCCTAA	GCCTCCTTCATGGCGTAGTT
acox1	GCACGGCTATTCTCACAGCA	ATCAAGAACCTGGCCGTCTG
cpt1a	ATCAAGAAGTGCCGGACGAG	AAGAGCCGAGTCATGGAAGC
acadm	AAAAGAGCCTGGGAACTCGG	CCATACGCCAACTCTTCGGT
sod2	GAGAGCAGCGGTCGTGTAAA	AGCCTCGTGGTACTTCTCCT

hmox1	GAACCCAGTCTATGCCCCAC	GGCGTGCAAGGGATGATTTC
sod1	TTCTCGTCTTGCTCTCTCTGG	CTTCTGCTCGAAGTGGATGGT
gpx1	AAGGCTCACCCGCTCTTTAC	GCACACCGGAGACCAAATGA
slc27a2	GCTACCACAGAAGTCGCTGA	GGTACTCCGCGATGTGTTGA
ppara	TCCAAGGGGGTGGCTACATA	CACTGGGCTACATCCTCGAC
pparg	ATTGAGTGCCGAGTCTGTGG	TCCGGCAGTTAAGATCACACC
dgat2	GAACGCAGTCACCCTGAAGA	CCCAGGAACCCTCCTCAAAG
gpam	TGGGCATCTCGTATGATCGC	TTCTGATAACGCCTCTCGCC
dgat1	TACCTAGTGAGCGTTCCCCT	TTGGAAGAATCGGCCCACAA
mtt	CTGCTATATGATGTTGTCAGCC	AGGAGATGTGTAGCCACTGC
srebp1c	CCCGGCTATTCCGTGAACAT	AGAACTCCCTGTCTCCGTCA
scd1	CGAGGGCTTCCACAACTACC	AACTCAGAAGCCCAAAGCTCA
acly	TCCCGTTCAGCTTCACACAA	GGATACAATGGTCCCAGCCC
fasn	GGCCCCTCTGTTAATTGGCT	GGATCTCAGGGTTGGGGTTG
acaca	GGGTGGTTCTTGGGTTGTGA	CGACGCATGGTTTTCACCAG
alb	CCCACTAGCCTCTGGCAAAA	ACACACCCCTGGAAAAAGCA
pdgfa	ATGTGAGGTGAGATGAGCCG	GGAGGAGAACAAAGACCGCA
sox9	CACAAGAAAGACCACCCCGA	CTCCGCTTGTCCGTTCTTCA
eln	TGCTGATCCTCTTGCTCAAC	TAATAGACTCCACCGGGAACT
hnf4a	GCAGTCAAGGCTCAGGAGTT	TCCGCAAAGCCATCAAGAGT
hnf1b	ATAGCTCCAACCAGACGCAC	TGTAGCGCACTCCTGACATC
acta2	TGAGCGTGGCTATTCCTTCG	AGCGTTCGTTTCCAATGGTG
thy1	AAGTCGGAACTCTTGGCACC	CCAGGCGAAGGTTTTGGTTC
clec4f	GATTGGGCTCACTGACCAGG	GTCTTCTCGCTCTCCGTTCC
cd36	TGCAGGTCTATCTACGCTGTG	TGTCTGGATTCTGGAGGGGT

Table S2. Primary antibodies used in the present work.

Antibody	Host species	Company	Catalogue number
GAPDH	Rabbit	ORIGENE	TA373085
NLRP3	Mouse	proteintech	68102-1-lg
GSDMD	Rabbit	proteintech	20770-1-AP
Caspase-1	Rabbit	ORIGENE	TA383869S
IL-1β	Rabbit	ORIGENE	TA3845202
IL-18	Rabbit	proteintech	10663-1-AP
beta IV Tublin	Mouse	ORIGENE	TA503129
STING	Rabbit	ORIGENE	TA385419S
cGAS	Rabbit	proteintech	29958-1-AP

Table S3. Baseline characteristics of participants in NHANES 2013–2018.

Characteristics	Mean (SD) or n (%)
Glyphosate, ng/mL	0.49 (0.58)
Age, year	50.05 (17.47)
Urinary creatinine, mg/100 mL	122.12 (79.33)
ALT, U/L	24.39 (17.98)
AST, U/L	24.49 (14.66)
GGT, U/L	28.71 (32.79)
ALP, IU/L	70.92 (23.25)
TB, umol/L	0.56 (0.30)
TP, g/dL	7.12 (0.45)
ALB, g/L	4.21 (0.34)
Gender, %	
Males	1817 (49.7)
Females	1837 (50.3)
Ethnicity, %	
Mexican American	526 (14.4)
Non-Hispanic Black	712 (19.5)
Non-Hispanic White	1522 (41.7)
Other Hispanic	360 (9.9)
Other Race - Including Multi-Racial	534 (14.6)
Family poverty income ratio, %	
Low	751 (20.6)
High	2903 (79.4)
Education, %	
9-11th grade (Includes 12th grade with no diploma)	408 (11.2)
College graduate or above	903 (24.7)
High school graduate/GED or equivalent	827 (22.6)

Less than 9th grade	286 (7.8)
Some college or AA degree	1230 (33.7)
BMI, %	
< 25 kg/m^2	1014 (27.8)
25–30 kg/m^2	1137 (31.1)
≥ 30 kg/m^2	1503 (41.1)
Cotinine, %	
At or above the detection limit	2429 (66.5)
Below lower detection limit	1225 (33.5)
Alcohol status, %	
No	803 (22.0)
Yes	1851 (78.0)
Physical Activity, %	
Moderate	789 (21.6)
No	2021 (55.3)
Vigorous	844 (23.1)
Cardiovascular disease, %	
No	3243 (88.8)
Yes	411 (11.2)

Abbreviations: ALT, alanine aminotransferase; AST, aspartate aminotransferase; ALP, alkaline phosphatase; GGT, gamma-glutamyl transferase; ALB, albumin; TP, total protein; TB, bilirubin; BMI, body mass index

Median (IQR) glyphosate: 0.32 (0.14–0.57) ng/mL

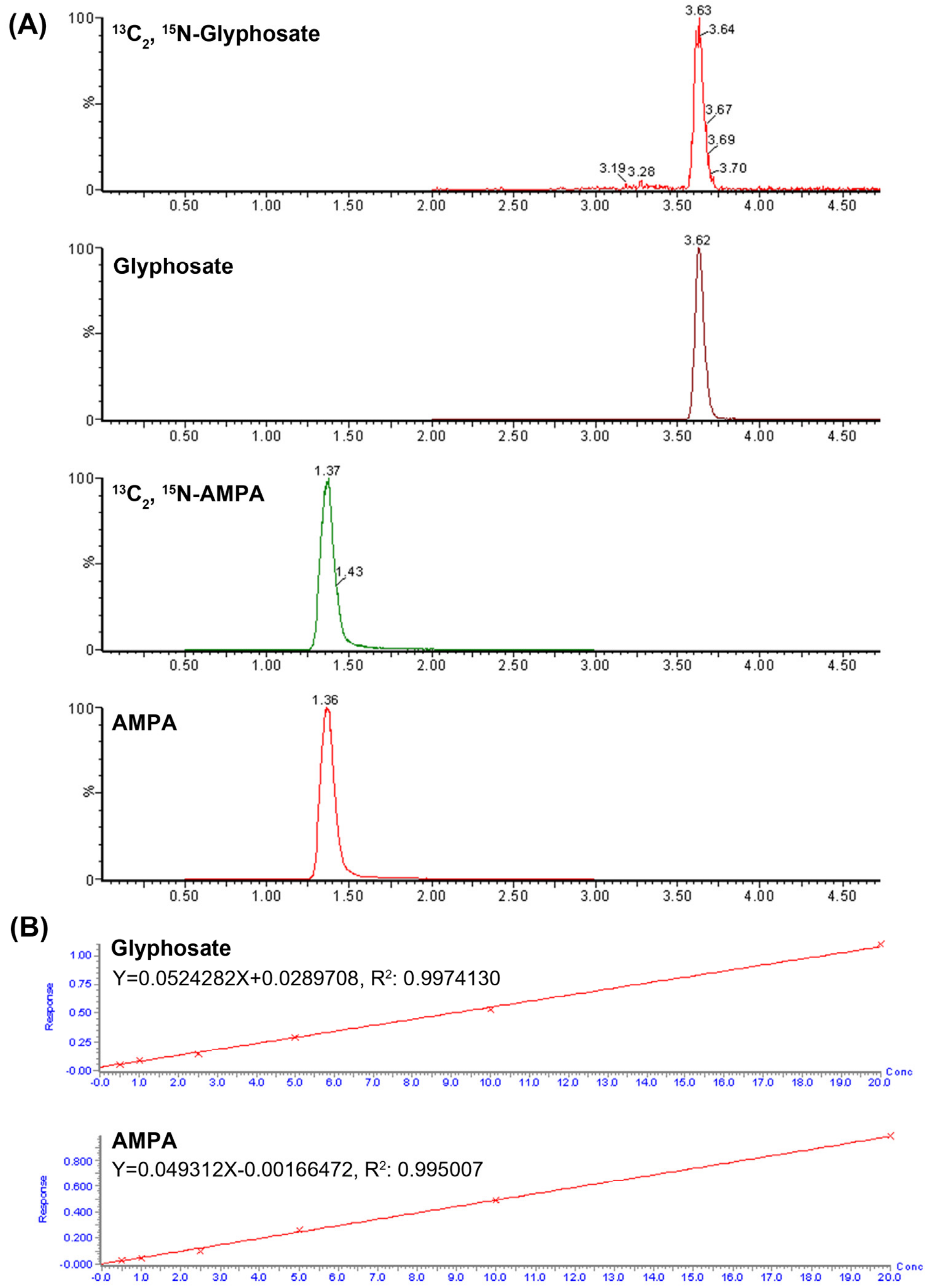

Figure S1. Quantitative LC-MS/MS analysis of glyphosate and AMPA.

(**A**) Chromatogram of glyphosate and AMPA with their respective internal standards. (**B**) Calibration curves for glyphosate and AMPA.

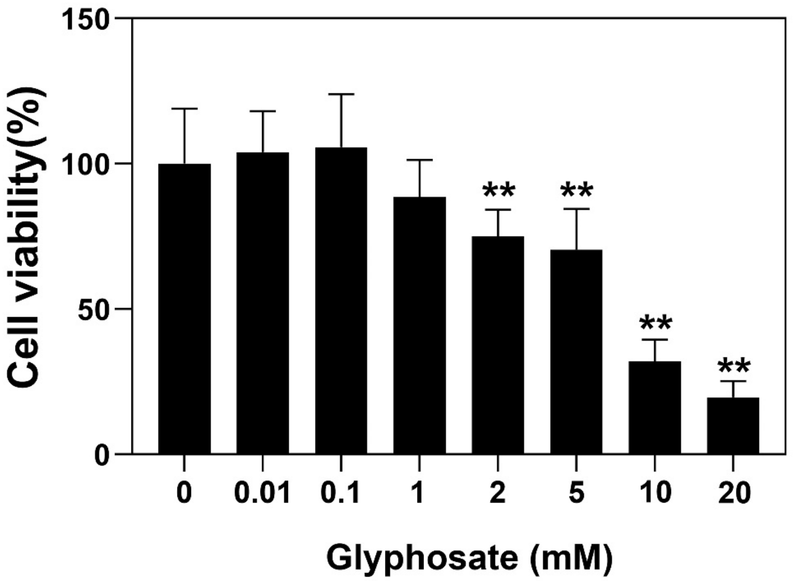

Figure S2. Assessment of RAW264.7 cell viability following glyphosate exposure. RAW264.7 cells were seeded in 96-well plates and exposed to glyphosate (0–20 mM) for 24 hours. Cell viability was assessed using the CCK-8 assay. Data are expressed as mean ± SD (n = 6). $^{**}P < 0.01$, compared with the control (0 mM).

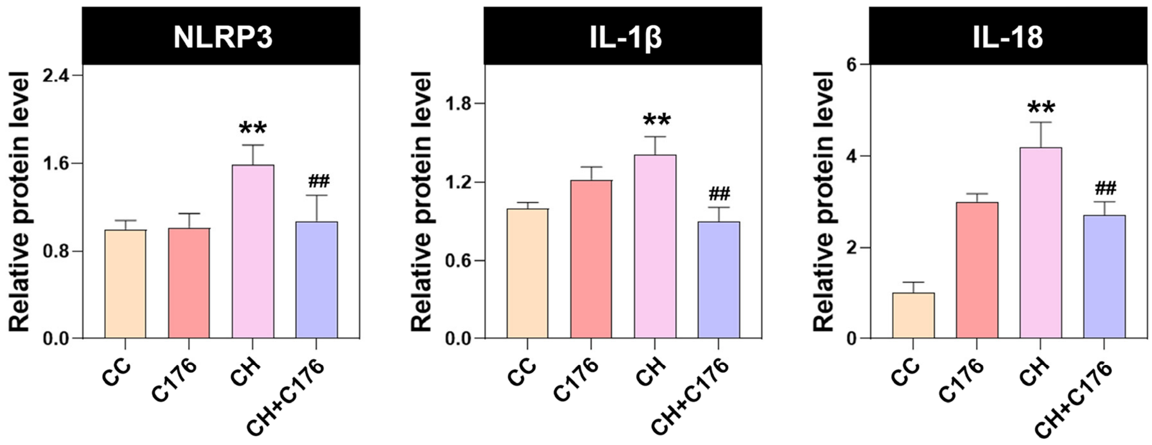

Figure S3. Quantification of NLRP3-related proteins after glyphosate and C176 treatment. Data are expressed as mean ± SD (n = 3). $^{**}P < 0.01$, compared with the control group (CC); ## $P < 0.01$, compared with the glyphosate group (CH).

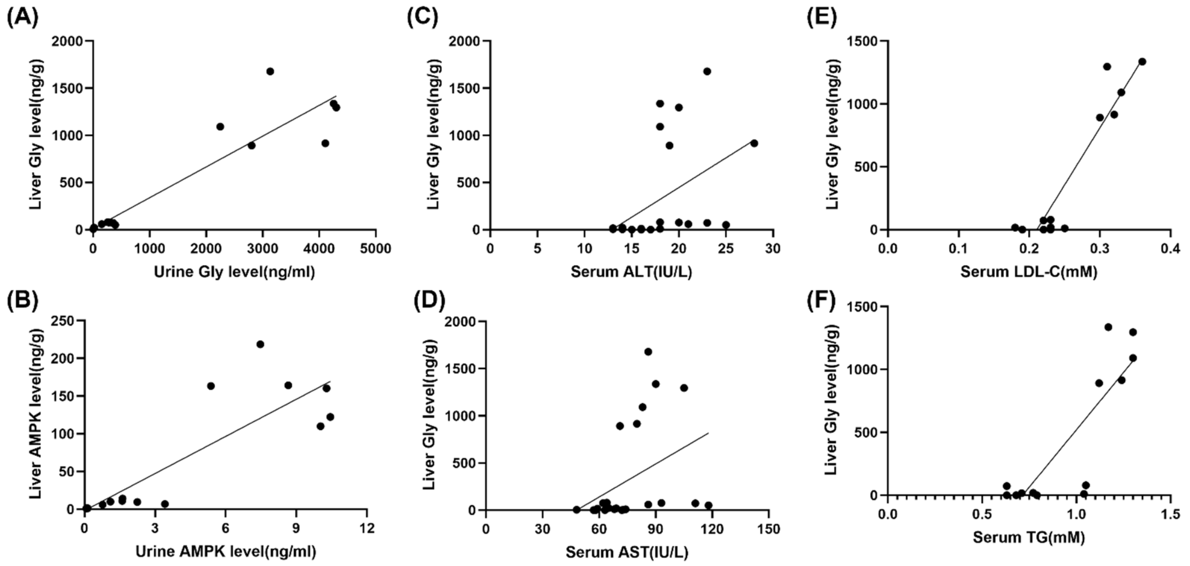

Figure S4. Spearman correlation between urine/serum compound and liver Gly (Glyphosate) /AMPK content.

(A) Spearman correlation between urine Gly and liver Gly content. (B) Spearman correlation between urine AMPK and liver AMPK content. (C) Spearman correlation between serum ALT and liver Gly content. (D) Spearman correlation between serum AST and liver Gly content. (E) Spearman correlation between serum LDL-C and liver Gly content. (F) Spearman correlation between serum TG and liver Gly content.

(A)

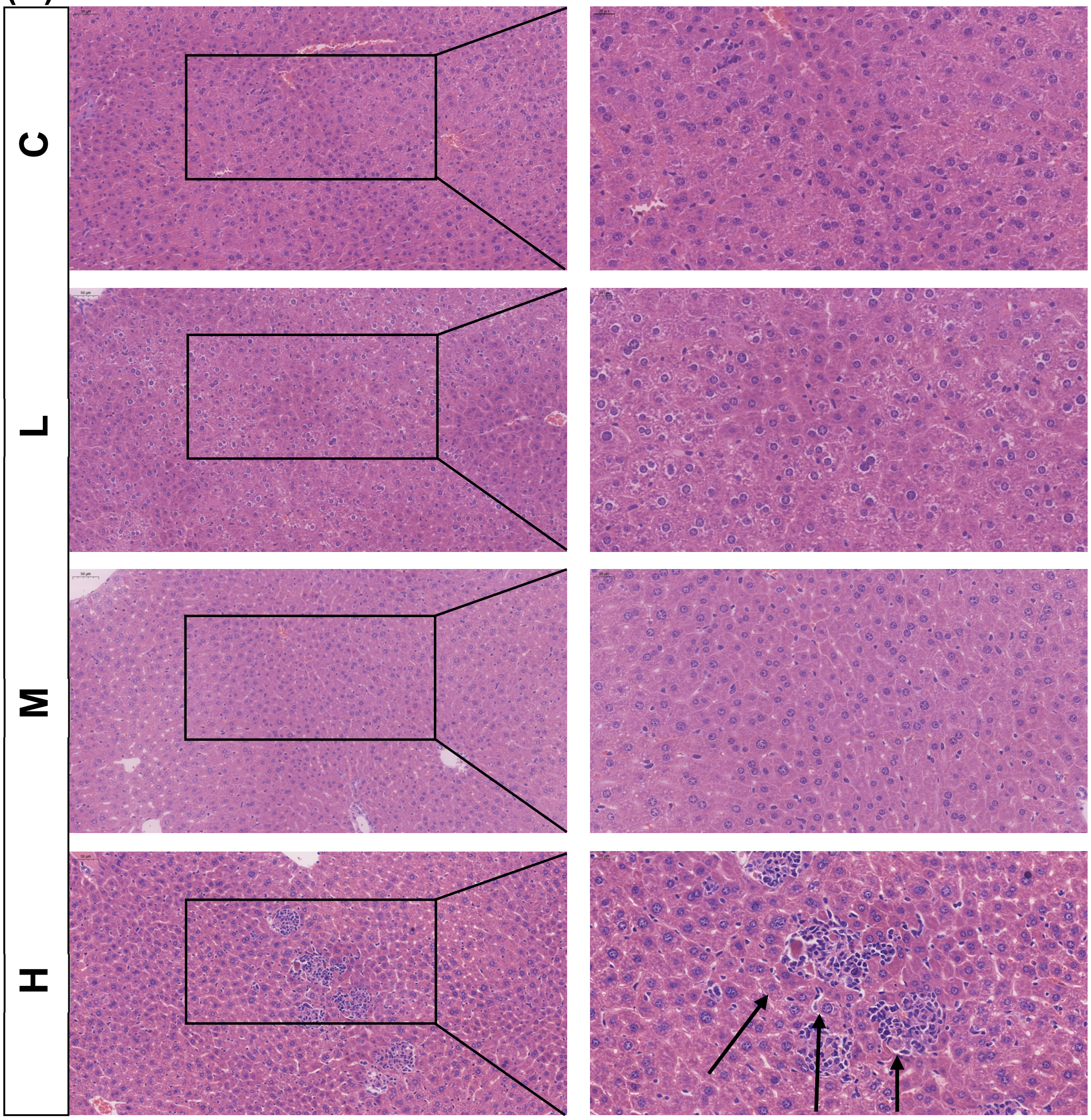

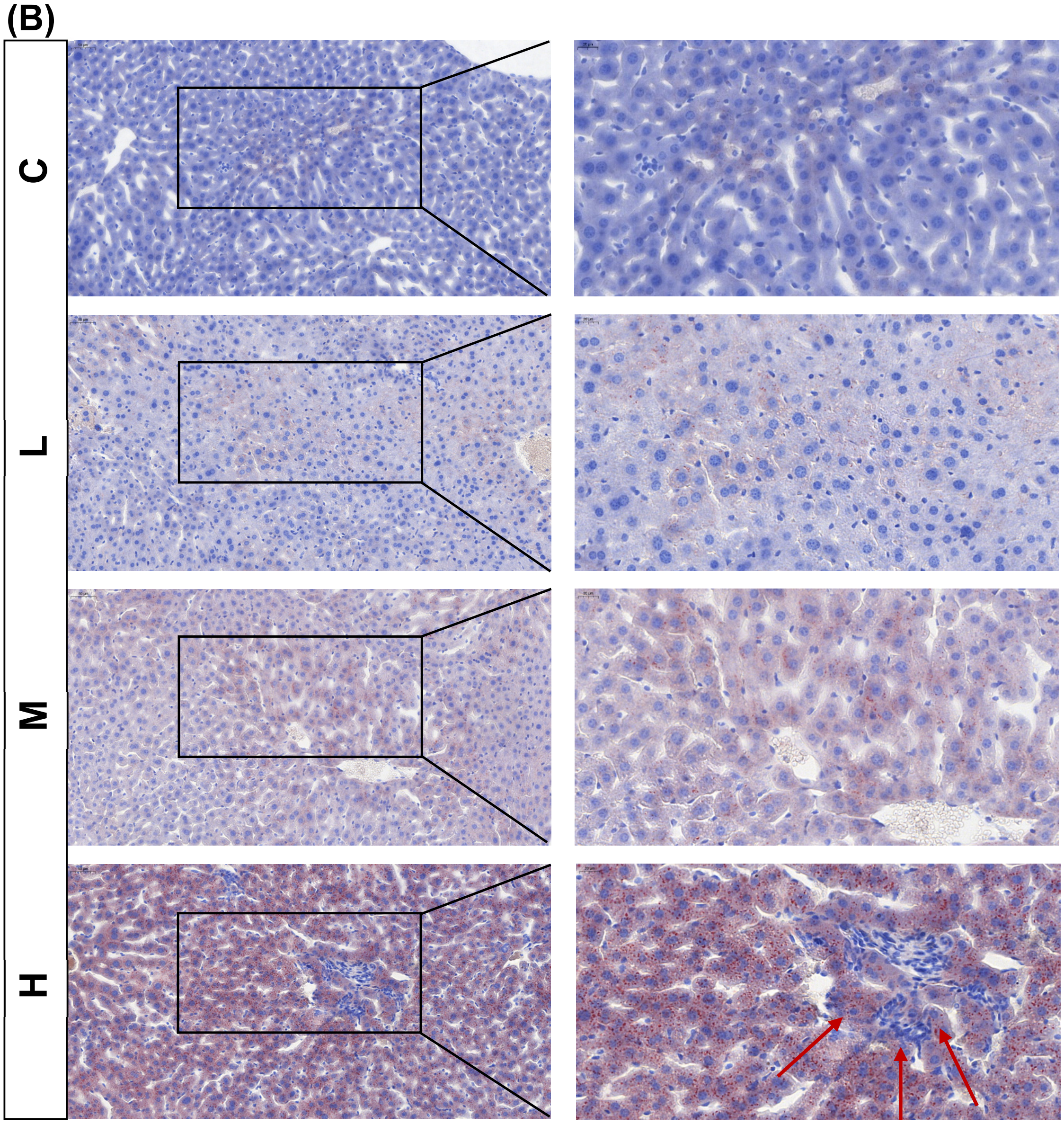

Figure S5. Representative images of liver sections stained with (A) H&E and (B) Oil Red O. Scale bars: 50 µm (top panel); 20 µm (bottom panel, magnified view). Black arrows indicate inflammatory infiltration. Red arrows indicate the presence of lipid droplets.

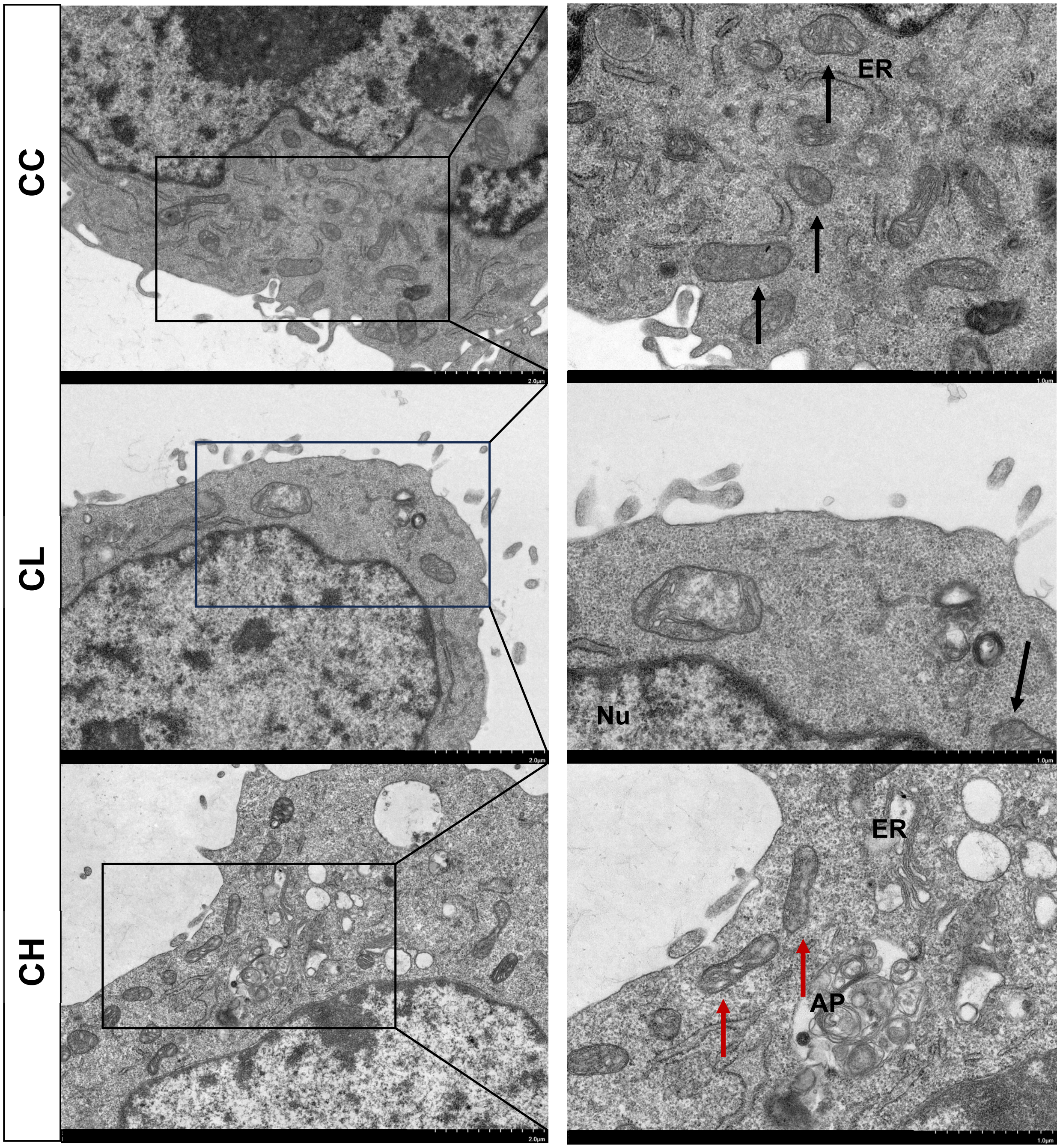

Figure S6. Mitochondrial ultrastructure in RAW264.7 cells was assessed by TEM.

Nu, nuclear; ER, endoplasmic reticulum; AP, autophagosome. Scale bars: 1 μm (top panel); 0.5 μm (bottom panel, magnified view). Black arrows indicate normal mitochondria; red arrows indicate impaired mitochondria: membrane lysis, along with disarray and fragmentation of the cristae.
